# Circulating Hepatitis B Virus (HBV) RNA and Conventional Markers in Treatment-Naive Persons With HBV in Senegal

**DOI:** 10.1093/infdis/jiaf190

**Published:** 2025-04-19

**Authors:** Lorin Begré, Messan K Akotia, Bruce S Wembulua, Melissa Pandi, Ousseynou Ndiaye, Judicaël Tine, Pascal Bittel, Christoph Niederhauser, Martin Stolz, Andri Rauch, Ndeye Fatou Ngom, Adrià Ramírez Mena, Gilles Wandeler, Moussa Seydi, for the Senegalese Hepatitis B Cohort Study (SEN-B), Amady Ndiaye, Oumy Camara, Aminata Diallo, Astou Diop, Boly Niang, Bintou Fall, Bruce Wembulua, Kevine Tiogouo, Marianne Berthé, Ndeye Maguette Fall, Adrià Ramírez Mena, Judicaël Tine, Aboubakar Sidikh Badiane, Fatima Sall, Daye Ka, Gilles Wandeler, Moussa Seydi, Ahmadou Mboup, Alassane Ndiaye, Abibatou Diaw, Assietou Gaye, Betty Fall, Houleye Saou, Ababacar Niang, Bessoum Sy, Sadio Ba, Astou Diagne, Khaly Diaw, Mamadou Gueye, Kiné Ndiaye, Ndeye Fatou Ngom, Mbaye Thiam, Ibrahima Niang, Joseph Diouf, Ndoye Diop, Aissatou Niang, Mariama Diedhiou, Khady Ndaw, Khady Ghassama, Khadim Faye, Fatou Diop, Marie Massaly, Aminata Ndoye Badji, Melissa Sandrine Pandi, Hubert Akotia, Bineta Seck Fall, Ousseynou Ndiaye, Albert Gautier Ndione, Marie-Joseph Dieme, Abdoulaye Keita, Louise Fortes, Fatou Fall, Adrià Ramírez Mena, Gilles Wandeler, Ibrahima Gueye, Ndeye Fatou Nguirane, Boubacar Coulibaly

**Affiliations:** Department of Infectious Diseases, Inselspital, Bern University Hospital, University of Bern, Bern, Switzerland; Centre Régional de Recherche et de Formation à la Prise en Charge Clinique, Fann University Hospital, Dakar, Senegal; Service des Maladies Infectieuses et Tropicales, Fann University Hospital, Dakar, Senegal; Centre Régional de Recherche et de Formation à la Prise en Charge Clinique, Fann University Hospital, Dakar, Senegal; Centre Régional de Recherche et de Formation à la Prise en Charge Clinique, Fann University Hospital, Dakar, Senegal; Service des Maladies Infectieuses et Tropicales, Fann University Hospital, Dakar, Senegal; Institute for Infectious Diseases, University of Bern, Bern, Switzerland; Institute for Infectious Diseases, University of Bern, Bern, Switzerland; Interregional Blood Transfusion Swiss Red Cross, Bern, Switzerland; Interregional Blood Transfusion Swiss Red Cross, Bern, Switzerland; Department of Infectious Diseases, Inselspital, Bern University Hospital, University of Bern, Bern, Switzerland; Centre de Traitement Ambulatoire, Fann University Hospital, Dakar, Senegal; Department of Infectious Diseases, Inselspital, Bern University Hospital, University of Bern, Bern, Switzerland; Service des Maladies Infectieuses et Tropicales, Fann University Hospital, Dakar, Senegal; Graduate School for Health Sciences, University of Bern, Bern, Switzerland; Department of Infectious Diseases, Inselspital, Bern University Hospital, University of Bern, Bern, Switzerland; Service des Maladies Infectieuses et Tropicales, Fann University Hospital, Dakar, Senegal; Institute of Social and Preventive Medicine, University of Bern, Bern, Switzerland; Service des Maladies Infectieuses et Tropicales, Fann University Hospital, Dakar, Senegal

**Keywords:** HBV RNA, hepatitis B virus, Africa, biomarkers, liver fibrosis

## Abstract

**Background:**

Hepatitis B virus (HBV) infection affects approximately 10% of the general population in West Africa. Circulating HBV RNA may help improve the characterization of HBV disease and prognosis. We aimed to evaluate the associations between HBV RNA and conventional biomarkers of HBV replication in the Senegalese Hepatitis B Cohort Study (SEN-B).

**Methods:**

We included all treatment-naive, human immunodeficiency virus–negative participants of SEN-B with chronic HBV infection confirmed by a quantitative hepatitis B surface antigen (HBsAg) >0.05 IU/mL. We quantified HBV RNA, HBV DNA, and HBsAg levels and evaluated associations between those markers stratified by HBV infection phase, alanine aminotransferase level, and liver fibrosis stage.

**Results:**

Of 719 participants, 17 (2.4%) were hepatitis B e antigen (HBeAg)-positive (EP), 620 (86.2%) were classified as HBeAg-negative chronic infection (ENCI), and 82 (11.4%) were classified as HBeAg-negative chronic hepatitis (ENCH). HBV RNA was undetectable in 361 (49.8%) participants, and detectable but unquantifiable in 188 (26.1%). HBV RNA levels correlated moderately with HBV DNA levels in EP (ρ = 0.58, *P* = .01) and ENCH (ρ = 0.54, *P* < .001) and weakly in ENCI (ρ = 0.39, *P* < .001). HBsAg levels were only significantly correlated with HBV RNA levels in the ENCH group (ρ = −0.22, *P* = .05). In multivariable logistic regression, HBV RNA levels and HBV RNA to HBV DNA ratio were independently associated with significant liver fibrosis.

**Conclusions:**

In our cohort of treatment-naive persons with HBV from Senegal, approximately 50% had undetectable HBV RNA levels. HBV RNA levels correlated with HBV DNA but not HBsAg levels in all phases of HBV infection and may provide an additional tool to assess HBV disease phase and activity.

## Teaser

Hepatitis B virus (HBV) RNA may improve the characterization of HBV disease and prognosis. In our cohort of treatment-naive persons with HBV from Senegal, HBV RNA levels correlated with HBV DNA but not hepatitis B surface antigen levels in all phases of HBV infection.

Worldwide, >250 million persons are living with a chronic hepatitis B infection [1]. In West Africa, the majority of infections occur at birth or during early childhood, affecting up to 10% of the population. Moreover, it is the leading cause of liver cirrhosis and hepatocellular carcinoma (HCC) [2]. In Senegal, >95% of persons with HBV (pwHBV) are hepatitis B e antigen (HBeAg) negative, and only a small number meet the criteria for antiviral therapy based on current European Association for the Study of the Liver (EASL) guidelines [3]. However, 1 in 8 pwHBV has significant liver fibrosis [4, 5].

Chronic HBV infection is a dynamic disease and pwHBV are typically classified into 4 phases of disease [5]. These phases are characterized by differences in HBV DNA and alanine aminotransferase (ALT) levels as well as HBeAg status, but these markers are often insufficient to determine HBV disease phase [6]. Current US and European guidelines recommend a liver biopsy to better characterize individuals when routine biomarkers are inconclusive [5, 7]. However, liver biopsies are invasive and not available in all clinical settings. Therefore, novel biomarkers including circulating HBV RNA may be needed to determine HBV disease phase.

Circulating HBV RNA in serum is mainly pregenomic RNA, the template for reverse transcription into HBV DNA, although other RNA subspecies can also be observed [8, 9]. Serum HBV RNA levels generally amount to 0.1%–1% of HBV DNA levels in untreated pwHBV and have been found to correlate closely with the transcriptional activity of the covalently closed circular DNA (cccDNA) pool, regardless of HBeAg status [8–10]. In individuals on antiviral therapy, persistently high HBV RNA levels are associated with higher degrees of liver fibrosis and HCC development [11, 12]. Thus, HBV RNA levels may help assess the intrahepatic HBV reservoir and predict progression of HBV-related liver disease. HBV RNA profiles according to the different HBV disease phases have recently been described in pwHBV from Hong Kong and North America as well as in a cohort from France and Italy [10, 13, 14]. However, whether HBV RNA levels are directly correlated with liver fibrosis and inflammation remains poorly understood in treatment-naive persons, and data from sub-Saharan Africa are lacking. We aimed to evaluate the association between circulating HBV RNA and conventional biomarkers of HBV replication in the Senegalese Hepatitis B Cohort Study (SEN-B) [15].

## MATERIALS AND METHODS

### Study Population and Design

We conducted a cross-sectional analysis including all treatment-naive participants of SEN-B who were human immunodeficiency virus (HIV) negative and had chronic HBV infection confirmed by a quantitative hepatitis B surface antigen (qHBsAg) level >0.05 IU/mL. Participants who were already on antiviral therapy at study inclusion or who did not have a plasma sample drawn prior to starting antiviral therapy were excluded. The SEN-B cohort includes individuals aged 18 years or older with a confirmed positive hepatitis B surface antigen (HBsAg) test followed at the Infectious and Tropical Disease Service and the Ambulatory Treatment Centre at Fann University Hospital, Dakar, Senegal [15]. The study was conducted in accordance with the Declaration of Helsinki. The Senegalese National Health Research Ethics Committee at the Health and Social Action Ministry of Senegal approved the SEN-B cohort (0061/MSAS/DPRS/CNERS) and all participants provided written informed consent.

### Study Procedures and Laboratory Analyses

Demographic, clinical, and laboratory data of all participants were assessed at study enrollment. We measured HBV DNA using a commercial quantitative nucleic acid test (cobas/TaqMan, Roche Diagnostic Systems, Meylan, France) with a lower limit of detection (LLOD) of 20 IU/mL. qHBsAg was measured using the e411 cobas HBsAg II assay (Roche Diagnostics, Rotkreuz, Switzerland), a commercial chemiluminescent microparticle immunoassay with a sensitivity of ≤0.05 IU/mL. HBV RNA was determined using the cobas HBV RNA automated investigational assay on the cobas 8800 system (Roche Molecular Diagnostics, Pleasanton, California, USA) with an LLOD <5 copies/mL and a linear range between 1 and 7 log_10_ copies/mL [16]. HBV RNA levels were categorized into 4 groups: <LLOD; LLOD to <1 log_10_ copies/mL; 1–3 log_10_ copies/mL; and >3 log_10_ copies/mL. A value of 0 copies/mL was arbitrarily assigned to HBV RNA levels <LLOD and a value of 0.78 log_10_ copies/mL to HBV RNA levels at the LLOD to <1 log_10_ copies/mL for statistical analyses. A single investigator determined liver stiffness using transient elastography according to the manufacturer's instructions (Fibroscan, Echosens, France) and we defined significant liver fibrosis as a liver stiffness measurement (LSM) >7.0–12.5 kPa and liver cirrhosis as LSM >12.5 kPa [17]. Severe liver steatosis was defined as a controlled attenuation parameter ≥280 decibels per meter (dB/m) [18]. ALT elevation was defined as ALT ≥30 IU/mL for male and ≥19 IU/mL for female participants, according to World Health Organization recommendations [17]. We further categorized ALT levels into 3 groups: ALT <1× the upper limit of normal (ULN); ALT 1 to <2× ULN; and ≥2× ULN. Alcohol consumption was assessed using Alcohol Use Disorders Identification Test–Consumption (AUDIT-C) and unhealthy alcohol consumption was defined as a score ≥4 for male and ≥3 for female participants [19].

### Definitions of Chronic Hepatitis B Phases

We classified participants into 3 phases of HBV infection: HBeAg-positive (EP) phase; HBeAg-negative chronic infection (ENCI) phase; and HBeAg-negative chronic hepatitis (ENCH) phase. Due to the very small number of persons with a positive HBeAg test at study inclusion, we classified them together, regardless of ALT and HBV DNA levels. However, as proposed by EASL and the American Association for the Study of Liver Diseases, HBeAg-negative participants with HBV DNA ≥2000 IU/mL along with elevated ALT and/or at least significant liver fibrosis (defined as LSM >7.0 kPa) were classified as being in the ENCH phase, whereas the remainder of participants was classified into the ENCI phase [5, 7].

### Statistical Analyses

We compared demographic and clinical characteristics of participants with and without detectable HBV RNA at study inclusion using descriptive statistics. We used χ^2^ tests or Fisher exact tests for categorical variables and Wilcoxon rank-sum tests for continuous variables, as appropriate. We described HBV DNA, qHBsAg, and HBV RNA levels in the different HBV infection phases and did pairwise comparisons between the phases. We evaluated associations between HBV RNA, HBV DNA, and qHBsAg levels using Spearman rank correlation coefficient stratified by HBV infection phase, ALT levels, and liver fibrosis stage. Using multivariable logistic regression, we investigated the association between LSM >7.0 kPa and HBV RNA levels, adjusted for preselected covariates including age, sex at birth, body mass index, HBeAg status, HBsAg levels, HBV DNA levels, ALT levels, unhealthy alcohol consumption, and liver steatosis. Similarly, we investigated the association between elevated ALT and HBV RNA levels using multivariable logistic regression models with the same preselected covariates except sex at birth, which was excluded because of the sex-dependent cut-offs included in the categorization of ALT levels. In the latter model, we replaced ALT levels by LSM >7.0 kPa as an additional variable. In exploratory analyses, we replaced HBV RNA and HBV DNA levels with HBV RNA to HBV DNA ratio (RNA/DNA ratio) in the multivariable logistic regression models evaluating the association with LSM >7.0 kPa and ALT elevation. We defined statistical significance as a *P* value <.05. All statistical analyses were performed using Stata/MP 16.0 software (StataCorp, College Station, Texas, USA).

## RESULTS

### Patient Characteristics

Of 918 participants included in SEN-B, we excluded 118 (12.9%) with HIV coinfection, 49 (5.3%) who were already on antiviral therapy at study inclusion, 1 (0.1%) without a plasma sample taken prior to tenofovir start, and 31 (3.4%) without a qHBsAg >0.05 IU/mL. Median age of the remaining 719 participants was 31 (interquartile range [IQR], 25–38) years, 341 (47.4%) were assigned female at birth, and 705 (98.1%) were born in Senegal. HBV RNA levels were undetectable in 361 (50.2%) participants, 188 (26.1%) had detectable but unquantifiable HBV RNA levels, 140 (19.5%) had HBV RNA levels between 1 and 3 log_10_ copies/mL, and 30 (4.2%) had HBV RNA levels >3 log_10_ copies/mL. Median HBV DNA was 2.8 (IQR, 2.1–3.4) log_10_ IU/mL, median qHBsAg was 3.8 (IQR, 3.3–4.1) log_10_ IU/mL, and median ALT was 18 (IQR, 13–24) IU/L. Significant liver fibrosis was present in 58 of 718 (8.1%) and liver cirrhosis in 18 (2.5%). In comparison to participants with undetectable HBV RNA, participants with detectable HBV RNA were more likely to be HBeAg positive (3.9% vs 0.8%, *P* = .01), and had higher median HBV DNA (3.1 [IQR, 2.6–3.8] log_10_ IU/mL vs 2.4 [IQR, 1.6–2.9] log_10_ IU/mL; *P* < .001) and aspartate aminotransferase levels (21 [IQR, 16–27]  IU/L vs 20 [IQR, 16–24] IU/L; *P* = .01) (Table 1). At least significant liver fibrosis (LSM >7.0 kPa) was present in 49 of 357 (13.7%) participants with detectable HBV RNA levels compared to 27 of 361 (7.5%) with undetectable HBV RNA levels (*P* = .01).

**Table 1.** Characteristics of Participants, Stratified by Hepatitis B Virus RNA Levels

Characteristic	HBV RNA ≥LLOD	HBV RNA <LLOD	*P* Value
	(n = 358)	(n = 361)	
Age, y, median (IQR)	31 (25–37)	31 (25–39)	.48
Female sex	161/358 (45.0)	180/361 (49.9)	.19
BMI, kg/m^2^, median (IQR)	22.1 (19.6–25.2)	22.1 (19.9–25.6)	.36
Unhealthy alcohol consumption	9/348 (2.6)	5/352 (1.4)	.29
HBeAg positive	14/358 (3.9)	3/361 (0.8)	.01
Anti-HBe positive	339/358 (94.7)	354/361 (98.1)	.02
qHBsAg, log_10_ IU/mL, median (IQR)	3.8 (3.3–4.2)	3.7 (3.2–4.1)	.10
qHBsAg ≤1000 IU/mL	57/358 (15.9)	71/361 (19.7)	.19
HBV DNA, log_10_ IU/mL, median (IQR)	3.1 (2.6–3.8)	2.4 (1.6–2.9)	<.001
HBV DNA ≤20 IU/mL	16/358 (4.5)	73/361 (20.2)	<.001
HDV antibody positive	0/358 (0.0)	5/361 (1.4)	.06
HCV antibody positive	0/357 (0.0)	1/359 (0.3)	>.99
LSM, kPa, median (IQR)	5.2 (4.4–6.1)	4.9 (4.1–5.8)	.01
LSM >7.0 kPa	49/357 (13.7)	27/361 (7.5)	.01
CAP, dB/m, median (IQR)	182 (154–215)	187 (159–217)	.30
CAP ≥280 dB/m	13/355 (3.7)	13/361 (3.6)	.97
ALT, IU/L, median (IQR)	18 (13–25)	17 (13–24)	.13
ALT elevation	104/358 (29.1)	94/361 (26.0)	.37
AST, IU/L, median (IQR)	21 (16–27)	20 (16–24)	.01
AST elevation	135/358 (37.7)	110/360 (30.6)	.04
Platelets, 10^9^/L, median (IQR)	280 (240–329)	288 (246–342)	.19
Phase of HBV disease			<.001
HBeAg-positive	14/358 (3.9)	3/361 (0.8)	
HBeAg-negative chronic infection	281/358 (78.5)	339/361 (93.9)	
HBeAg-negative chronic hepatitis	63/358 (17.6)	19/361 (5.3)	

Data are presented as no./No. (%) unless otherwise indicated.

Abbreviations: ALT, alanine aminotransferase; Anti-HBe, hepatitis B e antibody; AST, aspartate aminotransferase; BMI, body mass index; CAP, controlled attenuation parameter; HBV, hepatitis B virus; HCV, hepatitis C virus; HDV, hepatitis D virus; IQR, interquartile range; LLOD, lower limit of detection; LSM, liver stiffness measurement; qHBsAg, quantitative hepatitis B surface antigen.

**Figure 1.**
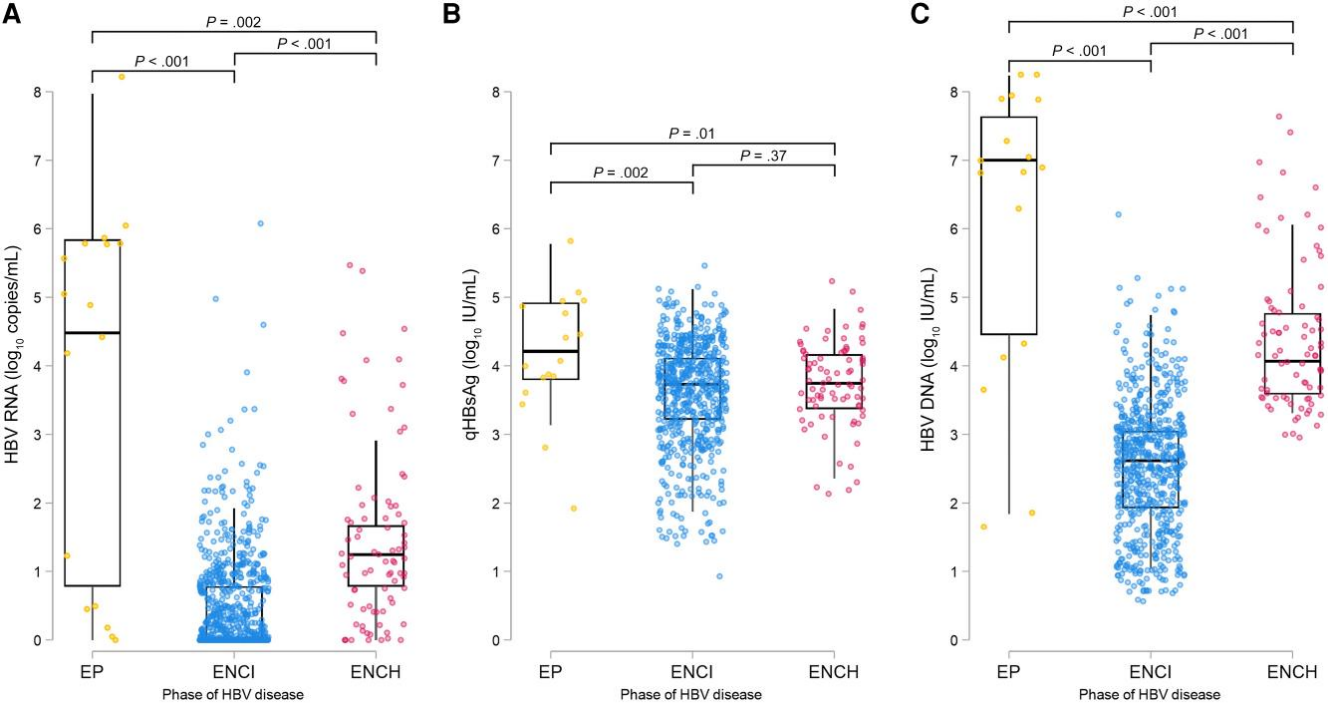
Hepatitis B virus (HBV) RNA (*A*), quantitative hepatitis B surface antigen (*B*), and HBV DNA (*C*) levels in the different phases of HBV infection. Boxes show median and interquartile range (IQR), whiskers show outliers until 1.5× IQR, and circles show individual values of participants. *P* values describe pairwise comparisons between groups. Abbreviations: ENCH, hepatitis B e antigen–negative chronic hepatitis; ENCI, hepatitis B e antigen–negative chronic infection; EP, hepatitis B e antigen-positive; HBV, hepatitis B virus; qHBsAg, quantitative hepatitis B surface antigen.

We classified 17 (2.4%) participants into the EP phase, 620 (86.2%) into the ENCI phase, and 82 (11.4%) into the ENCH phase. As depicted in Figure 1, EP participants had significantly higher median HBV DNA (7.0 [IQR, 4.5–7.6] log_10_ IU/mL), HBV RNA (4.5 [IQR, 0.8–5.8] log_10_ copies/mL), and qHBsAg levels (4.2 [IQR, 3.8–4.9] log_10_ IU/mL) compared to ENCI (HBV DNA 2.6 [IQR, 1.9–3.1] log_10_ IU/mL, *P* < .001; HBV RNA 0 [IQR, 0–0.8] log_10_ copies/mL, *P* < .001; qHBsAg 3.7 [IQR, 3.2–4.1] log_10_ IU/mL, *P* = .002) and ENCH participants (HBV DNA 4.1 [IQR, 3.6–4.8] log_10_ IU/mL, *P* < .001; HBV RNA 1.2 [IQR, 0.8–1.7] log_10_ copies/mL, *P* = .002; qHBsAg 3.7 [IQR, 3.4–4.2] log_10_ IU/mL, *P* = .01). Whereas participants in the ENCI phase had lower median HBV DNA (*P* < .001) and HBV RNA levels (*P* < .001) compared to ENCH participants, qHBsAg levels were similar in those 2 phases (*P* = .37). In addition, the highest RNA/DNA ratio was observed in EP participants and the lowest in ENCI participants. Detailed baseline characteristics according to HBV disease phase are shown in Supplementary Table 1.

### Correlation Between HBV RNA, HBV DNA, and qHBsAg

**Figure 2.**
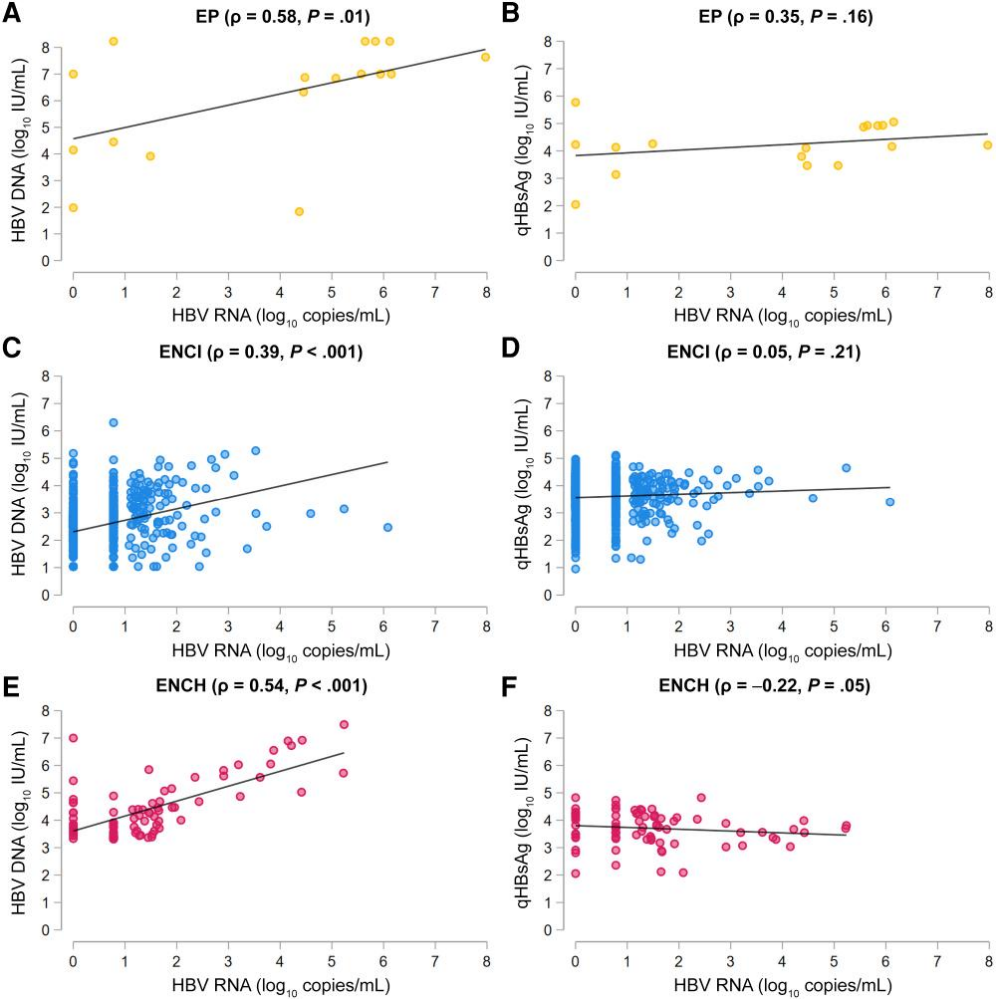
Correlation between hepatitis B virus (HBV) RNA and HBV DNA (*A*, *C*, *E*) and between HBV RNA and quantitative hepatitis B surface antigen (*B*, *D*, *F*) in the different phases of HBV infection. Abbreviations: ENCH, hepatitis B e antigen–negative chronic hepatitis; ENCI, hepatitis B e antigen–negative chronic infection; EP, hepatitis B e antigen-positive; HBV, hepatitis B virus; qHBsAg, quantitative hepatitis B surface antigen.

**Figure 3.**
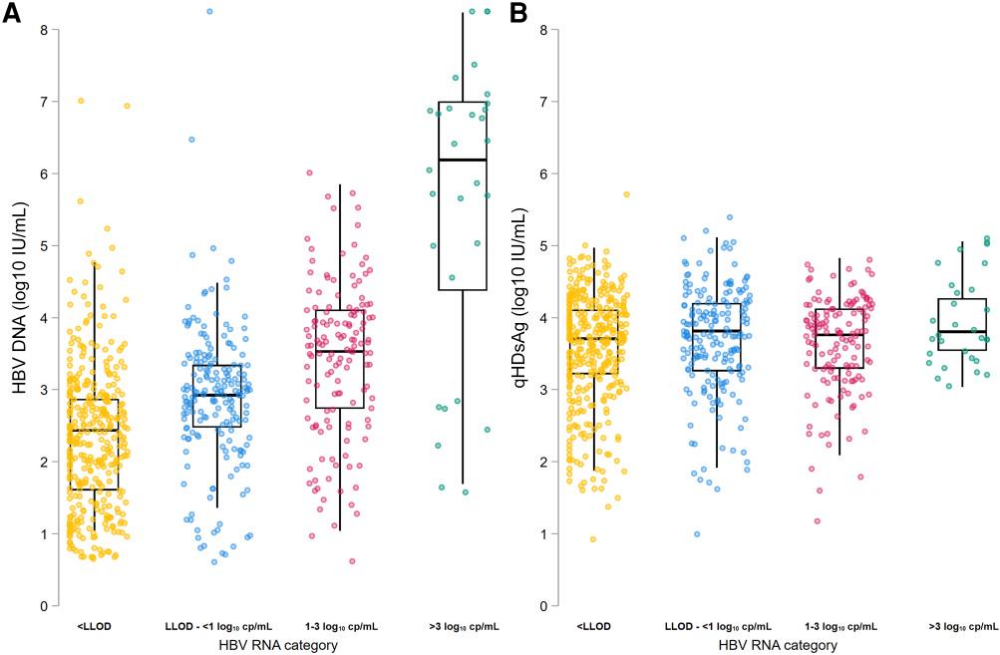
Hepatitis B virus (HBV) DNA levels (*A*) and quantitative hepatitis B surface antigen levels (*B*) according to HBV RNA categories. Abbreviations: cp/mL, copies per milliliter; HBV, hepatitis B virus; LLOD, lower limit of detection; qHBsAg, quantitative hepatitis B surface antigen.

HBV RNA levels correlated moderately with HBV DNA levels in EP (ρ = 0.58, *P* = .01) and ENCH participants (ρ = 0.54, *P* < .001) and weakly in ENCI participants (ρ = 0.39, *P* < .001) (Figure 2*A*, 2*C*, and 2*E*). We did not observe a correlation between HBV RNA and qHBsAg levels in EP (ρ = 0.35, *P* = .16) or ENCI participants (ρ = .05, *P* = .21) (Figure 2*B* and 2*D*), whereas we observed a weak but significant inverse correlation of these 2 markers in the ENCH phase (ρ = −0.22, *P* = .05; Figure 2*F*). We observed the highest HBV DNA levels among individuals with HBV RNA levels >3 log_10_ copies/mL, whereas lowest HBV DNA levels were observed among individuals with undetectable HBV RNA levels (*P* < .001; Figure 3*A*). qHBsAg levels did not differ between different categories of HBV RNA levels (*P* = .11; Figure 3*B*). HBV RNA levels correlated moderately with HBV DNA levels in participants with an LSM ≤7.0 kPa (ρ = 0.43, *P* < .001), and strongly in participants with significant fibrosis (>7–12.5 kPa, ρ = 0.65, *P* < .001) as well as participants with liver cirrhosis (>12.5 kPa, ρ = 0.78, *P* < .001; Supplementary Figure 1*A*, 1*C*, and 1*E*). We did not observe a correlation between HBV RNA and qHBsAg levels when stratifying according to liver fibrosis stage (all *P* > .15; Supplementary Figure 1*B*, 1*D*, and 1*F*). Similarly, HBV RNA levels correlated moderately to strongly with HBV DNA levels but not qHBsAg levels regardless of ALT category (Supplementary Figure 2).

### Association Between HBV RNA, Liver Fibrosis, and ALT Elevation

**Figure 4.**
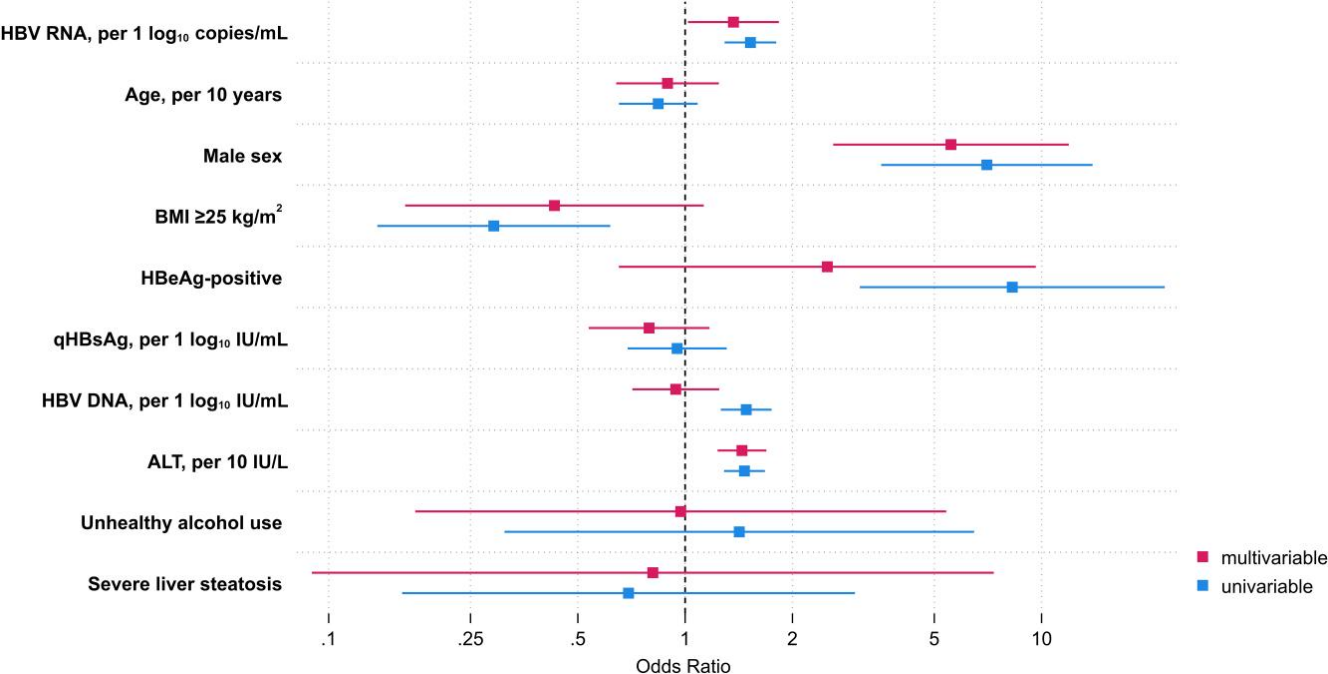
Univariable (blue) and multivariable (red) logistic regression models for factors associated with having at least significant liver fibrosis. Abbreviations: ALT, alanine aminotransferase; BMI, body mass index; HBeAg, hepatitis B e antigen; HBV, hepatitis B virus; qHBsAg, quantitative hepatitis B surface antigen.

Having at least significant liver fibrosis was associated with HBV RNA levels in multivariable logistic regression (odds ratio [OR], 1.37 [95% confidence interval {CI}, 1.02–1.83] per 1 log_10_ copies/mL increase). In addition, male sex at birth (OR, 5.57 [95% CI, 2.60–11.92]) and higher ALT levels (OR, 1.44 [95% CI, 1.23–1.69] per 10 IU/mL increase) were associated with LSM >7.0 kPa). HBV DNA and HBeAg status were associated with LSM >7.0 kPa in univariable logistic regression, but not after adjusting for covariates including HBV RNA (Figure 4, Supplementary Table 2). In the exploratory model including RNA/DNA ratio, LSM >7.0 kPa was associated with RNA/DNA ratio (OR, 2.44 [95% CI, 1.04–5.72]), male sex, higher ALT levels, and being HBeAg positive (Supplementary Table 2).

HBV RNA levels were also associated with elevated ALT levels in univariable logistic regression (OR, 1.23 [95% CI, 1.07–1.42]), but not after correcting for covariates (OR, 1.06 [95% CI, .87–1.29]) (Supplementary Table 3). However, being overweight (OR, 2.01 [95% CI, 1.33–3.03]), having at least significant liver fibrosis (OR, 1.94 [95% CI, 1.13–3.33]), higher qHBsAg levels (OR, 1.37 [95% CI, 1.05–1.78]), and higher HBV DNA levels (OR, 1.26 [95% CI, 1.05–1.50]) were independently associated with elevated ALT. RNA/DNA ratio was not associated with elevated ALT levels in the alternative model including RNA/DNA ratio instead of HBV RNA and HBV DNA levels (Supplementary Table 3).

## DISCUSSION

In our study of 719 treatment-naive Senegalese pwHBV, >85% were in the ENCI phase. Approximately 50% had undetectable HBV RNA levels and only one-quarter had levels above the lower limit of quantification. HBV RNA levels were significantly correlated with HBV DNA levels in all phases of HBV infection. HBV RNA but not HBV DNA was independently associated with having at least significant liver fibrosis. Our findings suggest that HBV disease phase characterization could be improved by quantification of serum HBV RNA.

High HBV RNA levels were an independent predictor for having at least significant liver fibrosis, in line with a previous, smaller study including treatment-naive individuals with liver biopsies from China [20]. Per 1 log_10_ copies/mL increase in HBV RNA levels, participants were 1.4 times more likely to have at least significant liver fibrosis as determined by transient elastography. In contrast, HBV DNA levels were no longer independently associated with liver fibrosis after adjusting for HBV RNA. Interestingly, in our exploratory logistic regression model including RNA/DNA ratio instead of HBV RNA and HBV DNA levels, higher RNA/DNA ratio was also an independent predictor of at least significant liver fibrosis. These findings suggest that plasma HBV RNA, as marker of cccDNA transcriptional activity, and RNA/DNA ratio, as marker of reverse transcriptional efficiency, may be better predictors of liver disease progression than HBV viral load in the absence of antiviral therapy. Individuals with high cccDNA transcriptional activity may have a higher risk of liver disease progression as highlighted in previous studies showing a higher risk of HCC among pwHBV with high HBV RNA levels on antiviral therapy [12, 21, 22]. The impact of highly efficient reverse transcription (ie, low RNA/DNA ratio) on HCC risk has not yet been determined.

HBV RNA levels were detectable in half of the participants. This proportion is lower than what has been previously observed in studies conducted in North America, where two-thirds of the participants had HBV RNA levels above the quantification limit and from Hong Kong, where >95% of the participants had detectable HBV RNA levels [13, 14]. These differences can be partly explained by the much higher proportion of EP and ENCH participants in the latter studies. In our cohort, 18% of ENCI participants and 56% of ENCH participants had quantifiable HBV RNA levels, in line with results from a European study, which found quantifiable HBV RNA levels in 57% of ENCH and 14% of ENCI participants [10]. However, in that study HBV RNA was quantifiable in all EP participants, whereas in our study only 70.6% of EP participants had quantifiable HBV RNA levels. Differences with regard to the detectability and quantification of HBV RNA in serum may be explained by host demographic and genetic factors, as well as virological factors including different HBV genotypes and basal precore mutations [23]. In Senegal, genotypes A and E are predominant whereas in the 2 studies with higher proportions with quantifiable HBV RNA levels, HBV genotypes B and C were responsible for the majority of infections [13, 14, 24]. Alternatively, methodological factors regarding the sample material like multiple freeze-thaw cycles and dilution for the analysis, but also different standards of HBV RNA assays used, may explain the discrepancies. Whereas the studies from North America and Hong Kong used an assay developed by Abbott Diagnostics, our study and the study by Testoni et al [10] used the cobas HBV RNA assay from Roche Diagnostics [16, 25]. The latter assay has been designed to preferentially measure RNA transcripts derived from cccDNA and would probably not recognize transcripts from integrated viral sequences [10].

In line with previous studies, we found a significant, mostly moderate correlation between HBV RNA and HBV DNA levels across all phases of HBV disease as well as liver fibrosis stages [13, 14]. However, this correlation was weaker than observed by Ghany and colleagues [14]. In addition, we observed a weak correlation between qHBsAg and HBV RNA levels only in ENCH participants, whereas Ghany et al observed a correlation in all disease phases [14]. One explanation for this difference may be the longer duration of infection in our population with infection usually having occurred at birth or during early childhood whereas in North America, a larger proportion acquired HBV later in life through horizontal transmission [26]. With longer duration of chronic HBV infection, a larger proportion of HBV viral sequences may be integrated into the host genome and the correlation between HBV RNA and qHBsAg in serum will decrease as the majority of HBsAg derive from integrated DNA in HBeAg-negative individuals [27–29].

Our study is among the first in sub-Saharan Africa to assess the potential of HBV RNA in characterizing HBV disease. Building on a large general population cohort with systematic storage of plasma samples, we included >700 well-characterized pwHBV. The comprehensive assessment of clinical and virological data, including systematic transient elastography measurements performed by a single investigator, allowed us to obtain accurate estimates of HBV liver disease activity and inflammation using noninvasive procedures. However, the small number of HBeAg-positive individuals precluded their subclassification into chronic HBeAg-positive hepatitis or infection. We used a highly sensitive HBV RNA assay, which quantifies HBV RNA mainly derived from cccDNA with an LLOD <5 copies/mL. This assay provides reproducible measurements across a broad range of HBV genotypes including genotypes A and E, the main genotypes in Senegal [16, 24, 30]. However, the lack of standardization of available HBV RNA assays may have complicated comparisons of HBV RNA results between studies. Moreover, we did not perform liver biopsies and therefore could not directly correlate intrahepatic cccDNA levels and transcriptional activity with HBV disease phase and liver fibrosis stage. Whereas differences in HBV RNA measurements across studies may also be driven by host and viral genetic factors, we could not evaluate the impact of different HBV genotypes on HBV RNA levels and HBV disease activity.

In conclusion, our findings indicate that levels of circulating HBV RNA levels change during the natural course of HBV infection and may be a useful additional tool to assess HBV disease phase and activity. However, long-term follow-up will be needed to further determine the value of circulating HBV RNA levels to identify individuals at high risk of progression of HBV-related liver disease and HCC who would benefit from early antiviral treatment.

## Supplementary Material

jiaf190_Supplementary_Data

## Supplementary Data

Supplementary materials are available at *The Journal of Infectious Diseases* online (http://jid.oxfordjournals.org/). Supplementary materials consist of data provided by the author that are published to benefit the reader. The posted materials are not copyedited. The contents of all supplementary data are the sole responsibility of the authors. Questions or messages regarding errors should be addressed to the author.

## Notes

***Acknowledgments.*** We thank the entire SEN-B team and all of the participants for their acceptance of study participation. The SEN-B Study Group members are Amady Ndiaye, Oumy Camara, Aminata Diallo, Astou Diop, Boly Niang, Bintou Fall, Bruce Wembulua, Kevine Tiogouo, Marianne Berthé, Ndeye Maguette Fall, Adrià Ramírez Mena, Judicaël Tine, Aboubakar Sidikh Badiane, Fatima Sall, Daye Ka, Gilles Wandeler, and Moussa Seydi from the Service des Maladies Infectieuses et Tropicales (SMIT), Centre Hospitalier Universitaire National (CHUN) de Fann, Dakar, Senegal; Ahmadou Mboup, Alassane Ndiaye, Abibatou Diaw, Assietou Gaye, Betty Fall, Houleye Saou, Ababacar Niang, Bessoum Sy, Sadio Ba, Astou Diagne, Khaly Diaw, Mamadou Gueye, Kiné Ndiaye, and Ndeye Fatou Ngom from the Centre de Traitement Ambulatoire, CHUN de Fann, Dakar, Senegal; Mbaye Thiam, Ibrahima Niang, and Joseph Diouf from the Department of Radiology, CHUN de Fann, Dakar, Senegal; Ndoye Diop from the Department of Radiology, Centre Hospitalier Universitaire de Saint Louis, Saint Louis, Senegal; Aissatou Niang, Mariama Diedhiou, Khady Ndaw, and Khady Ghassama from the Centre Régional de Recherche et Formation Clinique de Fann (CRCF), CHUN de Fann, Dakar, Senegal; Khadim Faye, Fatou Diop, and Marie Massaly from the Department of Laboratory Medicine, SMIT, CHUN de Fann, Dakar, Senegal; Aminata Ndoye Badji, Melissa Sandrine Pandi, Hubert Akotia, Bineta Seck Fall, and Ousseynou Ndiaye from the Department of Laboratory Medicine, CRCF, CHUN de Fann, Dakar, Senegal; Albert Gautier Ndione from the Department of Sociology, Cheikh Anta Diop University, Dakar, Senegal; Marie-Joseph Dieme and Abdoulaye Keita from the Department of Pathology, CHUN de Fann, Dakar, Senegal; Louise Fortes from the Department of Infectious Diseases, Centre Hospitalier Dalal Jamm, Guediawaye, Senegal; Fatou Fall from the Department of Hepatology, Hôpital Principal de Dakar, Dakar, Senegal; Adrià Ramírez Mena and Gilles Wandeler from the Department of Infectious Diseases, Bern University Hospital, Bern, Switzerland; and Ibrahima Gueye, Ndeye Fatou Nguirane, and Boubacar Coulibaly from Safaara Hépatites patient's association in Senegal.

***Author contributions.*** L. B., M. K. A., B. S. W., A. R. M., G. W., and M. Se. designed the study. M. K. A., M. P., O. N., P. B., C. N., and M. St. performed the laboratory analyses. L. B. analyzed the data. L. B., G. W., and M. Se. prepared the first draft of the manuscript, which was revised by all coauthors. B. S. W., J. T., N. F. N., A. R. M., G. W., and M. Se. contributed to data acquisition. All authors contributed to the interpretation of the analyses and approved the final version of the manuscript.

***Data availability.*** Data are not publicly available.

***Disclaimer.*** The funders had no role in study design, data collection and analysis, preparation of the manuscript, or decision to submit the manuscript for publication.

***Financial support.*** This work was supported by the Swiss National Science Foundation (PP00P3_211025), the Swiss Cancer Research Foundation (KLS-4879–08–2019), and the National Cancer Institute (U54-CA-254565). Roche Diagnostics International Ltd provided grant support with an investigator-initiated grant for the purpose of this study.
