# Supplementary material for: Circulating Hepatitis B Virus (HBV) RNA and Conventional Markers in Treatment-Naive Persons With HBV in Senegal
Source: J Infect Dis. 2025 Apr 19;232(1):e64–72. doi: 10.1093/infdis/jiaf190 (PMC12308681; doi:10.1093/infdis/jiaf190)
Supplement: jiaf190_Supplementary_Data [file jiaf190_supplementary_data.docx]

**SUPPLEMENTARY MATERIAL**

# Table of Contents

[Supplementary Table 1: Baseline characteristics, stratified by phase of HBV infection. 2](#_Toc178250454)

[Supplementary Table 2: Factors associated with significant liver fibrosis, determined by transient elastography >7.0 kPa. 3](#_Toc178250455)

[Supplementary Table 3: Factors associated with elevated ALT, defined as ALT ≥30 IU/ml for men and ALT ≥19IU/ml for women 4](#_Toc178250456)

[Supplementary Figure 1: Correlation between HBV RNA and HBV DNA (A, C, E) and between HBV RNA and qHBsAg (B, D, F) according to degree of liver fibrosis 5](#_Toc178250457)

[Supplementary Figure 2: Correlation between HBV RNA and HBV DNA (A, C, E) and between HBV RNA and qHBsA (B, D, F) according to ALT levels 6](#_Toc178250458)

# Supplementary Table 1: Baseline characteristics, stratified by phase of HBV infection.

|  | **HBeAg-positive** | **HBeAg-negative chronic infection** | **HBeAg-negative chronic hepatitis** | **p-value** |
| --- | --- | --- | --- | --- |
|  | **N=17** | **N=620** | **N=82** |  |
| Age [years] | 27 (23-29) | 31 (25-39) | 30 (25-36) | 0.05 |
| Female sex | 5/17 (29.4%) | 296/620 (47.7%) | 40/82 (48.8%) | 0.32 |
| BMI [kg/m^2^] | 21.2 (20.2-24.0) | 22.2 (19.8-25.4) | 21.7 (19.3-25.6) | 0.78 |
| Unhealthy alcohol consumption | 0/17 (0.0%) | 11/605 (1.8%) | 3/78 (3.8%) | 0.44 |
| anti-HBe positive | 3/17 (17.6%) | 609/620 (98.2%) | 81/82 (98.8%) | <0.001 |
| qHBsAg [log_10_ IU/ml] | 4.2 (3.8-4.9) | 3.7 (3.2-4.1) | 3.7 (3.4-4.2) | 0.01 |
| qHBsAg ≤1,000 IU/ml | 1/17 (5.9%) | 118/620 (19.0%) | 9/82 (11.0%) | 0.09 |
| HBV DNA [log_10_ IU/ml] | 7.0 (4.5-7.6) | 2.6 (1.9-3.1) | 4.1 (3.6-4.8) | <0.001 |
| HBV DNA ≤20 IU/ml | 0/17 (0.0%) | 89/620 (14.4%) | 0/82 (0.0%) | <0.001 |
| HBV RNA [log_10_ copies/ml] | 4.5 (0.8-5.8) | 0.0 (0.0-0.8) | 1.2 (0.8-1.7) | <0.001 |
| HBV RNA category |  |  |  | <0.001 |
| <LLOD | 3/17 (17.6%) | 339/620 (54.7%) | 19/82 (23.2%) |  |
| ≥LLOD to <1 log_10_ copies/ml | 2/17 (11.8%) | 169/620 (27.3%) | 17/82 (20.7%) |  |
| 1 to 3 log_10_ copies/ml | 1/17 (5.9%) | 104/620 (16.8%) | 35/82 (42.7%) |  |
| >3 log_10_ copies/ml | 11/17 (64.7%) | 8/620 (1.3%) | 11/82 (13.4%) |  |
| log_10_ HBV RNA to HBV DNA ratio | 0.7 (0.2-0.8) | 0.0 (0.0-0.3) | 0.3 (0.2-0.4) | <0.001 |
| HDV antibody positive | 0/17 (0.0%) | 5/620 (0.8%) | 0/82 (0.0%) | >0.99 |
| HCV antibody positive | 0/16 (0.0%) | 1/619 (0.2%) | 0/81 (0.0%) | >0.99 |
| Liver stiffness measurement [kPa] | 6.5 (5.5-9.8) | 5.0 (4.2-5.8) | 5.6 (4.6-7.5) | <0.001 |
| LSM >7.0 kPa | 8/17 (47.1%) | 44/619 (7.1%) | 24/82 (29.3%) | <0.001 |
| Controlled attenuation parameter [dB/m] | 194 (161-206) | 184 (155-216) | 193 (160-219) | 0.66 |
| CAP ≥280 dB/m | 0/17 (0.0%) | 24/618 (3.9%) | 2/81 (2.5%) | 0.87 |
| ALT [IU/l] | 29 (20-46) | 17 (13-22) | 31 (22-40) | <0.001 |
| ALT elevation | 8/17 (47.1%) | 118/620 (19.0%) | 72/82 (87.8%) | <0.001 |
| AST [IU/l] | 33 (18-44) | 20 (16-24) | 28 (21-38) | <0.001 |
| AST elevation | 10/16 (62.5%) | 175/620 (28.2%) | 60/82 (73.2%) | <0.001 |
| Platelets [10^9^/l] | 225 (204-274) | 287 (249-338) | 271 (228-329) | <0.001 |

*Data are presented as median (IQR) for continuous measures, and n/total (%) for categorical measures. Abbreviations: ALT, alanine aminotransferase; anti-HBe, hepatitis B e antibody; AST, aspartate aminotransferase; BMI, body mass index; CAP, controlled attenuation parameter; kPA, kilopascal; LLOD, lower limit of detection; LSM, liver stiffness measurement; qHBsAg, quantitative HBsAg.*

# Supplementary Table 2: Factors associated with significant liver fibrosis, determined by transient elastography >7.0 kPa.

|  |  | **Unadjusted** | | **Adjusted (N = 690)** | | | |
| --- | --- | --- | --- | --- | --- | --- | --- |
|  |  |  | | **with HBV DNA and HBV RNA levels** | | **with HBV RNA to HBV DNA ratio** | |
|  | N | **OR (95% CI)** | **p-value** | **OR (95% CI)** | **p-value** | **OR (95% CI)** | **p-value** |
| Male sex | 718 | 7.02 (3.55-13.91 | <0.001 | 5.57 (2.60-11.92) | <0.001 | 5.66 (2.66-12.04) | <0.001 |
| Age, per 10 years | 718 | 0.84 (0.65-1.08) | 0.18 | 0.89 (0.64-1.24) | 0.50 | 0.90 (0.65-1.25) | 0.55 |
| BMI ≥25 kg/m^2^ | 711 | 0.29 (0.14-0.62) | 0.001 | 0.43 (0.16-1.13) | 0.09 | 0.41 (0.15-1.06) | 0.07 |
| Unhealthy alcohol use | 699 | 1.42 (0.31-6.47) | 0.65 | 0.97 (0.17-5.40) | 0.97 | 0.96 (0.17-5.51) | 0.97 |
| Severe liver steatosis | 716 | 0.69 (0.16-2.99) | 0.62 | 0.81 (0.09-7.34) | 0.85 | 0.77 (0.09-6.99) | 0.82 |
| ALT, per 10 IU/l increase | 718 | 1.47 (1.29-1.67) | <0.001 | 1.44 (1.23-1.69) | <0.001 | 1.46 (1.25-1.71) | <0.001 |
| HBeAg-positive | 718 | 8.27 (3.09-22.15) | <0.001 | 2.51 (0.65-9.63) | 0.18 | 3.52 (1.07-11.58) | 0.04 |
| qHBsAg, per log_10_ IU/ml increase | 718 | 0.95 (0.69-1.31) | 0.75 | 0.79 (0.54-1.17) | 0.24 | 0.80 (0.55-1.18) | 0.26 |
| HBV DNA, per log_10_ IU/ml increase | 718 | 1.48 (1.26-1.75) | <0.001 | 0.94 (0.71-1.25) | 0.67 |  |  |
| HBV RNA, per log_10_ copies/ml increase | 718 | 1.52 (1.29-1.80) | <0.001 | 1.37 (1.02-1.83) | 0.04 |  |  |
| HBV RNA to HBV DNA ratio | 718 | 2.1 (1.22-4.00) | 0.01 |  |  | 2.44 (1.04-5.72) | 0.04 |

*Abbreviations: ALT, alanine aminotransferase; BMI, body mass index; qHBsAg, quantitative HBsAg.*

# Supplementary Table 3: Factors associated with elevated ALT, defined as ALT ≥30 IU/ml for men and ALT ≥19IU/ml for women

|  |  | **Univariable models** | | **multivariable models (N=690)** | | | |
| --- | --- | --- | --- | --- | --- | --- | --- |
|  |  |  | | **with HBV DNA and HBV RNA levels** | | **with HBV RNA to HBV DNA ratio** | |
|  | N | **OR (95% CI)** | **p-value** | **OR (95% CI)** | **p-value** | **OR (95% CI)** | **p-value** |
| Age, per 10 years | 719 | 1.09 (0.93-1.27) | 0.31 | 1.11 (0.92-1.34) | 0.26 | 1.12 (0.93-1.34) | 0.24 |
| BMI ≥25 kg/m^2^ | 712 | 1.64 (1.15-2.35) | 0.01 | 2.01 (1.33-3.03) | 0.001 | 1.92 (1.28-2.88) | 0.002 |
| Unhealthy alcohol use | 700 | 1.05 (0.33-3.39) | 0.93 | 0.80 (0.21-3.03) | 0.75 | 0.83 (0.22-3.13) | 0.79 |
| Significant liver fibrosis | 718 | 1.96 (1.20-3.20) | 0.01 | 1.94 (1.13-3.33) | 0.02 | 2.17 (1.28-3.68) | 0.004 |
| Severe liver steatosis | 716 | 0.98 (0.40-2.36) | 0.96 | 0.57 (0.21-1.57) | 0.28 | 0.57 (0.21-1.54) | 0.27 |
| HBeAg-positive | 719 | 2.40 (0.91-6.30) | 0.08 | 0.71 (0.21-1.57) | 0.58 | 1.63 (0.57-4.64) | 0.36 |
| qHBsAg, per log_10_ IU/ml increase | 719 | 1.35 (1.07-1.71) | 0.01 | 1.37 (1.05-1.78) | 0.02 | 1.47 (1.14-1.90) | 0.003 |
| HBV DNA, per log_10_ IU/ml increase | 719 | 1.33 (1.17-1.51) | <0.001 | 1.26 (1.05-1.50) | 0.01 |  |  |
| HBV RNA, per log_10_ copies/ml increase | 719 | 1.23 (1.07-1.42) | 0.004 | 1.06 (0.87-1.29) | 0.57 |  |  |
| HBV RNA to HBV DNA ratio | 719 | 1.17 (0.72-1.92) | 0.53 |  |  | 1.12 (0.66-1.92) | 0.68 |

*Abbreviatons: ALT, alanine aminotransferase; BMI, body mass index; qHBsAg, quantitative HBsAg.*

# Supplementary Figure 1: Correlation between HBV RNA and HBV DNA (A, C, E) and between HBV RNA and qHBsAg (B, D, F) according to degree of liver fibrosis

Abbreviations: ENCH, HBeAg-negative chronic hepatitis; ENCI, HBeAg-negative chronic infection; EP, HBeAg-positive phase; kPA, kilopascal; LSM; liver stiffness measurement; qHBsAg, quantitative HBsAg.

# Supplementary Figure 2: Correlation between HBV RNA and HBV DNA (A, C, E) and between HBV RNA and qHBsAg (B, D, F) according to ALT levels

*Abbreviations: ALT, alanine aminotransferase; ULN, upper limit of normal; qHBsAg, quantitative HBsAg.*
